# Sieve element occlusion provides resistance against *Aphis gossypii* in TGR‐1551 melons

**DOI:** 10.1111/1744-7917.12610

**Published:** 2018-07-18

**Authors:** Hsuan‐Chieh Peng, Gregory P. Walker

**Affiliations:** ^1^ Department of Microbiology and Plant Pathology University of California Riverside California USA; ^2^ Department of Entomology University of California Riverside California USA

**Keywords:** aphid feeding behavior, *Aphis gossypii*, *Cucumis melo*, insect–plant resistance, phloem occlusion, sieve element occlusion proteins

## Abstract

Feeding behavior and plant response to feeding were studied for the aphid *Aphis gossypii* Glover on susceptible and resistant melons (cv. Iroquois and TGR‐1551, respectively). Average phloem phase bout duration on TGR‐1551 was <7% of the duration on Iroquois. Sixty‐seven percent of aphids on TGR‐1551 never produced a phloem phase that attained ingestion (EPG waveform E2) in contrast to only 7% of aphids on Iroquois. Average bout duration of waveform E2 (scored as zero if phloem phase did not attain E2) on TGR‐1551 was <3% of the duration on Iroquois. Conversely, average bout duration of EPG waveform E1 (sieve element salivation) was 2.8 times greater on TGR‐1551 than on Iroquois. In a second experiment, liquid nitrogen was used to rapidly cryofix leaves and aphids within a few minutes after the aphids penetrated a sieve element. Phloem near the penetration site was then examined by confocal laser scanning microscopy. Ninety‐six percent of penetrated sieve elements were occluded by protein in TGR‐1551 in contrast to only 28% in Iroquois. Usually in TGR‐1551, occlusion was also observed in nearby nonpenetrated sieve elements. Next, a calcium channel blocker, trivalent lanthanum, was used to prevent phloem occlusion in TGR‐1551, and *A. gossypii* feeding behavior and the plant's phloem response were compared between lanthanum‐treated and control TGR‐1551. Lanthanum treatment eliminated the sieve element protein occlusion response and the aphids readily ingested phloem sap from treated plants. This study provides strong evidence that phloem occlusion is a mechanism for resistance against *A. gossypii* in TGR‐1551.

## Introduction

Most aphids (Aphididae) are obligate phloem sap‐feeders (Miles, 1987, 1999). To feed, they penetrate a phloem sieve element with their stylet tips and depend on the phloem functioning as a pipeline to provide a continuous flow of sap through the penetrated sieve element. The continuous flow allows them to feed from the same sieve element for hours or even days at a time (Tjallingii, 1995). In order to maintain this continuous flow, aphids must keep the sieve element alive and functioning and avoid triggering a phloem occlusion response or reverse occlusion if it occurs.

Plants have two well‐known mechanisms of phloem occlusion that prevent loss of sap when a sieve element is damaged—one based on callose, a β‐1,3 glucan polymer, and the other based on proteins. Callose shuts down the flow of sap by deposition in the sieve pores (the perforations on the sieve plates that allow sap to flow from one sieve element to the next), essentially choking off the pores and reducing or stopping the flow of sap from one sieve element to the next (Eschrich, 1975). The deposition of callose in the sieve pores requires *de novo* synthesis (Xie *et al*., 2011) and thus is relatively slow and can take 10 min or longer to reach maximum deposition (Furch *et al*., 2007, 2010). Deposition of callose is reversible if the damage is not too severe (Furch *et al*., 2007, 2010).

In response to damage, sieve element occlusion (SEO) proteins also plug sieve pores and prevent loss of sap. SEO proteins are constitutively present in translocating sieve elements and thus do not require *de novo* synthesis to respond to damage; consequently plugging by SEO proteins is extremely rapid (within a few seconds) (Knoblauch *et al*., 2001). As is the case for callose deposition, if damage is not too severe, the protein occlusions dissipate and flow of sap can resume (Knoblauch *et al*., 2001; Furch *et al*., 2007, 2010; Medina‐Ortega & Walker, 2013). SEO proteins are widespread and likely ubiquitous among dicotyledon plants (Rüping *et al*., 2010; Ernst *et al*., 2011). In most dicotyledonous plants, SEO proteins are believed to be filamentous and confined to a parietal position in translocating sieve elements (Ehlers *et al*., 2000; Froleich *et al*., 2011; Anstead *et al*., 2012; Pagliari *et al*., 2017). Upon damage, they are released into the lumen of the sieve element and are carried to the downstream sieve plate where they occlude the sieve pores (Ehlers *et al*., 2000; Ernst *et al*., 2012; Jekat *et al*., 2013). SEO proteins of the Fabaceae are unique; they form discrete structures, called forisomes, that reside in the lumen of the sieve element (Pélissier *et al*., 2008; Rüping *et al*., 2010). In translocating sieve elements, forisomes are compact and spindle‐shaped and do not obstruct the flow of sap; however, in response to damage, they rapidly swell to a size that occludes the sieve element, stopping the flow of sap (Knoblauch *et al*., 2001, 2012). The genus *Cucurbita* in the Cucurbitaceae has two additional unrelated soluble proteins, phloem protein 1 and 2 (PP1 and PP2), that circulate in the phloem sap and in response to damage, contribute to occluding the sieve pores (Clark *et al*., 1997; Golecki *et al*., 1999).

It has been hypothesized many times over the past two decades that one function of protein and callose plugs is to deter phloem sap‐feeding insects such as aphids and whiteflies (Cole, 1994; Girousse & Bournoville, 1994; Caillaud *et al*., 1995a,b; Caillaud & Niemeyer, 1996; Klingler *et al*., 1998, 2005; Sauge *et al*., 1998; Garzo *et al*., 2002; Cardoza *et al*., 2005; Tjallingii, 2006; Jiang & Walker, 2007; Zhu *et al*., 2011; Lightle *et al*., 2012). Many of these studies compared the feeding behavior of aphids on susceptible and resistant plants and found that penetration of a sieve element on a susceptible plant is usually followed by a short (ca. 30–60 s) bout of salivation, which is subsequently followed by a long bout of sap ingestion; whereas on resistant plants, sieve element penetration is often followed by very long periods of salivation, and the stylets are frequently withdrawn from the sieve element without engaging in sap ingestion or are withdrawn after only a short bout of sap ingestion. It was hypothesized in these studies that sieve element penetration on resistant plants triggers phloem occlusion, preventing the insects from making a successful transition from salivation to phloem sap ingestion.

The first experimental data demonstrating that SEO protein plugging can be induced by aphids and impair their feeding were in a pair of papers examining the feeding behavior of a specialist aphid and a generalist aphid on faba bean, *Vicia faba*. Faba bean forisomes (Fabaceae SEO proteins) did not respond to sieve element penetration by the faba bean specialist, *Acyrthosiphon pisum* (Harris), and the aphids readily engaged in long bouts of phloem sap ingestion shortly after initial penetration of the sieve element (Walker & Medina‐Ortega, 2012). In contrast, when the generalist aphid, *Myzus persicae* Sulzer, penetrated sieve elements of faba bean (which is a suboptimal host for *M. persicae*), the forisomes expanded, occluding the sieve elements, and the aphids were unable to ingest phloem sap in over 90% of sieve element penetrations (Medina‐Ortega & Walker, 2015). Forisome occlusion is triggered by a calcium influx into the sieve element (Knoblauch *et al*., 2001; Furch *et al*., 2009) and can be prevented with a calcium channel blocker such as lanthanum (Furch *et al*., 2009). When forisome occlusion was prevented with lanthanum, inhibition of ingestion was alleviated and *M. persicae* readily engaged in long bouts of sap ingestion on faba bean, providing direct evidence that forisome occlusion was likely responsible for *M. persicae*’s difficulty ingesting phloem sap on faba bean (Medina‐Ortega & Walker, 2015).

The experiments of Medina‐Ortega and Walker compared the SEO response of the same plant to two aphid species, for which the plant is either the primary host or a suboptimal host. Similar experiments comparing the SEO response of a resistant and susceptible plant variety to the same aphid would be even more relevant for determining the role of SEO proteins in crop variety resistance. Consequently, this study was conducted to compare the SEO response of two melon (*Cucumis melo* L.) lines to feeding by the aphid *Aphis gossypii* Glover: a susceptible commercial variety, Iroquois, and a highly resistant line, TGR‐1551. TGR‐1551 was chosen based on prior work by Garzo *et al*. (2002) and Tjallingii (2006) that demonstrated *A. gossypii* had difficulty ingesting phloem sap on TGR‐1551—most penetrations of sieve elements were terminated without ingesting phloem sap and were characterized by very long periods of salivating into the phloem, the same as *M. persicae* feeding on faba bean as observed by Medina‐Ortega & Walker (2015). Recently, Garzo *et al*. (2018) found coagulations in the stylet food canal of *A. gossypii* feeding on TGR‐1551 and lumps of electron‐dense material around the maxillary stylet tips in the sieve element in contrast to clear food canals and sieve elements in aphids feeding on a susceptible melon variety. The main objectives of the present study were to (i) verify the results of Garzo *et al*. (2002) that phloem sap ingestion by *A. gossypii* (in this case a North American population) is inhibited on TGR‐1551, (ii) determine if sieve element penetration by *A. gossypii* triggers SEO protein occlusion on TGR‐1551 but not on a susceptible melon variety, and (iii) determine if inhibition of sap ingestion on TGR‐1551 is eliminated when SEO protein occlusion is prevented by a calcium channel blocker.

## Materials and methods

### Aphids and plants

Two cultivars of melon, *Cucumis melo*, were grown in potted soil (University of California soil mix III) in a greenhouse: cv. Iroquois (Sustainable Seed Company, Chico, California, USA) and TGR‐1551 (seeds provided by the United States Department of Agriculture Nationa1 Germplasm Collection). The *Aphis gossypii* colony was started from aphids collected from squash near Reedley, California about a decade ago and reared on various melon varieties since then. During the experiments, they were reared on Iroquois, a variety on which they flourished.

### Electrical penetration graphs

Electrical penetration graphs (EPGs) were recorded in a Faraday cage using Giga‐8 and Giga‐4 EPGs (EPG Systems, Wageningen, the Netherlands), DI‐710 and DI‐720 analog‐to‐digital interfaces, and Windaq software (A–D interfaces and software from Dataq Instruments, Akron, Ohio, USA). The analog‐to‐digital conversion rate was 100 samples/s/channel. Adult apterate and last instar *A. gossypii* nymphs were used. A 10–20 mm length of thin gold wire, 12.5 µm diameter (Sigmund Cohn Corp., Mt. Vernon, New York, USA) was attached to the dorsum of the aphids with water soluble silver glue (4 mL glue; Ross glue stick, Ross Adhesives, Toronto, Ontario, Canada, 4 mL water, 4 g silver flake; 8–10 µm average size, Inframat Advanced Materials, Manchester, Connecticut, USA, and one drop of Triton X‐100).

EPGs were recorded from aphids feeding on the abaxial side of young, mature melon leaves, intact on the plant. To record EPGs, the abaxial side of a leaf was strapped to a plastic “leaf holder” (described in detail by Walker & Medina‐Ortega, 2012) with strips of Parafilm (Bemis Flexible Packaging, Neenah, Wisconsin, USA). Each leaf holder had a 12.5 mm diameter “access hole” that allowed the wired aphid access to the abaxial side of the leaf. Each leaf holder also had a short (ca. 30 mm) copper wire that protruded over the access hole. A thin gold wire with an aphid attached at one end was glued by its opposite end to the copper wire using a conductive adhesive (Dag 503, Ladd Research Industries, Williston, Vermont, USA). The gold wire was long enough to allow the aphid to settle on the leaf exposed by the access hole. Once set up, EPGs were recorded by touching the input of the head stage amplifier to the copper wire. The leaf holder served to prevent the leaf from moving during EPG recording and made it easy to first put the aphid on the leaf with the abaxial side facing up and then when the insect got a firm grip on the leaf, turn the leaf over in its natural orientation with the abaxial side facing down. The leaf holder was held steady by an alligator clip on a metal stand that rested on the floor of the Faraday cage.

The experiments all focused on the “phloem phase” of feeding behavior. Phloem phase begins with penetration of a sieve element by the stylet tips and is characterized by two known behaviors represented by EPG waveform E1 (salivation into the sieve element) and waveform E2 (ingestion of phloem sap). Waveform E1 begins shortly after penetration of the sieve element and may be followed by E2 or may be terminated by withdrawing the stylets from the sieve element without engaging in sap ingestion.

### Cryofixation and confocal laser scanning microscopy

Two experiments, described below, recorded EPGs from aphids, and several minutes after the beginning of phloem phase, the aphids and leaf on which they were feeding were cryofixed with liquid nitrogen. This was done to fix the stylets *in situ* in the leaf so that the penetration site in the phloem could later be examined. Immediately before cryofixation, the nitrogen was cooled below its –196 °C boiling point by vacuum treatment (Froelich *et al*., 2011). After several minutes under vacuum, the nitrogen froze, lowering its temperature. Releasing the vacuum resulted in a return to the liquid state, but below its boiling point. The liquid nitrogen was poured onto the aphids and plants usually within *ca*. 15 s of releasing the vacuum. The leaf was then covered with powdered dry ice to keep it frozen. Following cryofixation, the tissue was freeze substituted in 95% ethanol and prepared for examination by confocal laser scanning microscopy (CLSM) using a similar procedure to that described by Walker and Medina Ortega (2012). The frozen leaves were severed from the plant and placed in 95% ethanol at dry ice temperature (–78.5 °C) and kept at this temperature for two or more days and then moved to –20 °C for one or more days. Samples were then removed from the –20 °C freezer and put in a styrofoam container where they slowly came to room temperature. The aphids usually broke away from their stylets during cryofixation, leaving the stylets in the leaf. To find the stylets, the freeze‐substituted samples were placed in a Petri dish with 95% ethanol, and the abaxial sides of the leaves (the sides on which the aphids were feeding) were examined with a Leica MZ16 stereomicroscope and substage lighting. Once the stylets were located, the leaf was turned over, and electrolytically sharpened tungsten wires (Brady, 1965) held by pin vices were used to carefully remove all plant tissues from the adaxial surface down to the phloem where the stylet tips were. This included removing the xylem. Dissections were done both free‐hand and with assistance of a Mk1 micromanipulator (Singer Instruments, Roadwater, UK). A razor blade was then used to cut out a small piece of the leaf (approximately 50 mm^2^) containing the dissected area, and this piece was placed, dissected side up, in a small Petri dish (35 mm diameter). Excess ethanol was removed, and several drops of the Molecular Probes (Invitrogen, Carlsbad, CA, USA) stain DiOC_7_(3) (0.1% in 95% ethanol) were placed on the samples. Lids were placed on the Petri dishes and the dishes were placed in a container with an ethanol‐saturated atmosphere to retard evaporation. Specimens were stained with DiOC_7_(3) for >30 min; overstaining was not a problem. The DiOC_7_(3) solution was then carefully blotted away and replaced by several changes of 95% ethanol. The 95% ethanol was then replaced with several changes of water followed by several drops of the Molecular Probes stain Sulforhodamine 101 (1% in water) for 1 h or more (again overstaining was not a problem). After staining, the sulforhodamine solution was blotted away and replaced by several changes of water. Samples were then transferred to a microscope slide with the dissected surface facing up. Water was then blotted off and slides were cover‐slipped using an aqueous mounting medium (SHUR/Mount Aqua‐Poly; Ted Pella, Redding, California, USA). Samples were examined with confocal microscopy (Zeiss 510, Leica SP2 and Leica SP5 microscopes were used) using the default settings for FITC/TRITC double labeling.

The cryofixation procedure was successful in approximately one out of two or three attempts in fixing the stylets *in situ* in the leaf. Success rate was better when the liquid nitrogen was poured onto the adaxial side of the leaf with the feeding aphid on the opposite side rather than pouring the liquid nitrogen directly on the aphid, which tended to knock the aphid off the leaf, pulling out the stylets. Consequently, almost all the cryofixations were done by pouring liquid nitrogen on the adaxial side with the aphid feeding on the downward‐facing abaxial side.

### Aphid feeding behavior on Iroquois and TGR‐1551 melons

EPGs were recorded from aphids feeding on Iroquois and TGR‐1551 in order to compare phloem phase behaviors between the two melons. The abaxial side of the leaf was turned up during EPG recordings. Each replicate used a different aphid and different plant. Analysis was restricted to only those aphids that produced phloem phase during the recording. Twenty‐seven aphids on Iroquois and 30 aphids on TGR‐1551 produced one or more phloem phases.

The total duration of each phloem phase and duration of each E2 in each phloem phase (recorded as zero if the phloem phase did not attain E2) were measured. The average durations of phloem phase and E2 were first calculated for each aphid (only aphids that produced at least one phloem phase were included), and then these averages were compared between Iroquois and TGR‐1551 using the nonparametric Wilcoxon rank sum test. In cases where phloem phase and E2 extended past the end of recording, their observed durations (time from their start to end of recording) were included in calculations. If phloem phase extended past the end of recording while the aphid was still in waveform E1, then E2 was scored as missing data because it is unknown whether or not the phloem phase would have eventually attained E2.

For the purposes of this study, the term “initial E1” is defined as the period from the voltage drop at the start of phloem phase to the beginning of E2 for phloem phases that produced E2, or the period from start of phloem phase to end of phloem phase for phloem phases that did not produce E2. Initial E1 duration was compared between Iroquois and TGR‐1551 as described above for the analysis of phloem phase and E2 durations. In cases where E1 extended past the end of recording, the observed duration (time from start of phloem phase to end of recording) was included in calculations.

The second experiment determined whether or not the penetrated sieve element and nearby sieve elements were occluded by SEO‐protein shortly after initiation of phloem phase in Iroquois and TGR‐1551. Aphids and the leaves on which they were feeding were instantaneously cryofixed as described previously. EPGs were recorded with the abaxial leaf surface facing up and then, in most cases, the leaf was turned over prior to cryofixation as described previously so that the liquid nitrogen would not dislodge the aphid from the leaf. Ninety‐one percent of the samples were cryofixed 2–10 min after the start of phloem phase (median = 3.5 min). A few were cryofixed at longer times after the start of phloem phase. Leaf tissue was then processed for examination by CLSM as described previously. Each replicate used a different plant and different aphid.

### Lanthanum experiments

Results of the previous experiment implicated SEO protein occlusion as the mechanism inhibiting phloem sap ingestion on TGR‐1551. To test this hypothesis, two experiments were conducted with aphids feeding on TGR‐1551 where SEO protein occlusion was prevented by the calcium channel blocker, trivalent lanthanum. In each experiment, midribs of young mature leaves of TGR‐1551 were treated with one of two saline solutions: standard physiological saline (10 mmol/L calcium chloride, 10 mmol/L potassium chloride and 5 mmol/L sodium chloride) or “lanthanum saline” (10 mmol/L lanthanum chloride, 10 mmol/L potassium chloride and 5 mmol/L sodium chloride). In a similar previous study (Medina‐Ortega & Walker, 2015), the treatments were injected in faba bean leaves with a hypodermic needle; however, melon leaves were too thin to be injected, so an alternate method was used to apply the treatments. To facilitate penetration of the solutions to the phloem, the vascular core of the midrib was exposed from the adaxial side by dissecting away the epidermis and most of the vascular parenchyma overlying the xylem using an electrolytically sharpened tungsten needle (Brady, 1965). The outer mesophyll approximately 0.25 mm either side of the midrib also was removed. The dissected area of the midrib was approximately 15–20 mm in length and about 2/3 of the distance from the base to apex of the leaf. The tissue was covered with standard saline during dissection to prevent desiccation. After dissection, the dissected area was kept covered in standard saline for at least 110 min and then the standard saline was blotted away with tissue paper and replaced with either lanthanum saline or with fresh standard saline (lanthanum and control treatments, respectively). EPG recording began 2–4 h after this change of salines. One leaf per plant was treated in this manner with either lanthanum saline, or the control standard physiological saline; treated leaves were left attached to the plant.

For recording EPGs, the treated leaf of each plant was attached to a leaf holder, as described previously, with the 12.5 mm diameter hole positioned on the abaxial side of the leaf, where the aphids would feed, directly opposite the dissected section of the midrib on the adaxial side. After attaching the leaves to the leaf holder, fresh lanthanum or standard saline, depending on treatment, was applied to the dissected area and covered with a microscope cover glass (18 mm diameter) which was held in place with strips of Parafilm. This kept the saline solution in contact with the dissected area and prevented it from dripping off when the leaf was turned over.

The first experiment recorded EPGs from aphids feeding on lanthanum and control treatments in order to compare phloem phase behaviors between the two treatments. Each replicate used a different aphid and different plant. EPGs were recorded from aphids feeding on the abaxial side of the leaf with the abaxial side facing up to facilitate visual monitoring of the aphid. To ensure that the aphids were feeding on the section of the midrib that was affected by the two treatments (lanthanum or control), the aphids were kept on the treated section of the midrib; when they strayed from the treated section, they were moved back with a fine artist brush. Only phloem phases verified visually to be on the treated section were used in analysis. Analysis was restricted to only those aphids that produced one or more phloem phases during the recording. Seventeen aphids on lanthanum‐treated plants and 23 aphids on the control plants produced one or more phloem phases. Calculation of variables and analysis was the same as described previously for comparing Iroquois and TGR‐1551.

A second experiment was conducted to determine whether or not the penetrated sieve element and nearby sieve elements were occluded by P‐protein shortly after initiation of phloem phase in the lanthanum and control treatments. Within 3–6 min of beginning of phloem phase, the leaf and aphid were instantaneously cryofixed as described previously. Leaf tissue was then processed for examination by CLSM as described previously. Each replicate used a different plant and different aphid. As noted previously, freezing the stylets *in situ* in the leaf tissue works best if the liquid nitrogen is poured on the adaxial side on the leaf opposite where the aphid is feeding on the abaxial side. For this reason, the EPG recordings were done mostly with the abaxial side facing down so that the liquid nitrogen could be poured onto the adaxial side. However, the leaves had to be periodically turned over to the abaxial side facing up in order to move the aphids back to the treated section of the midrib if they strayed away. Once the aphids settled back on the treated section of the midrib, the leaves were turned over so the abaxial side faced down. For this experiment, a narrow inset was placed in the opening of the leaf holder (fig. S1C in supporting information in Walker & Medina‐Ortega, 2012) to help restrict movement of the aphid to the dissected area of the midrib. Only aphids visually verified to be feeding on the treated section of the midrib were cryofixed.

## Results

### Phloem phase behavior on TGR‐1551 and Iroquois

Duration of phloem phase differed greatly between aphids feeding on TGR‐1551 and Iroquois. 133 phloem phases from 30 aphids were recorded on TGR‐1551 and 40 phloem phases from 27 aphids on Iroquois. Phloem phase bout duration was significantly shorter on TGR‐1551 than on Iroquois (34.8 ± 49.7 vs. 511.6 ± 342.0, *P* < 0.0001, Wilcoxon rank sum test, Table 1). Duration of sieve element ingestion (waveform E2) per phloem phase (scored as zero if the phloem phase did not result in E2) also was significantly shorter on TGR‐1551 than on Iroquois (12.5 ± 44.8 vs. 496.9 ± 350.3, *P* < 0.0001, Wilcoxon rank sum test, Table 1). The distributions of the E2 data were skewed with long right hand tails (especially for TGR‐1551); therefore, the medians may provide a better statistic showing the degree of difference between TGR‐1551 and Iroquois: 0 min and 395 min, respectively (Table 1). For 20 out of 30 aphids (67%) on TGR‐1551, none of the phloem phases produced waveform E2 in contrast to only 2 out of 27 aphids (7%) on Iroquois (*P* < 0.0001, Fisher's Exact Test). Considering all phloem phase bouts (rather than compiled per insect), 114 out of 124 phloem phases (92%) on TGR‐1551 did not produce E2 (9 of the 133 phloem phases on Iroquois were not included in this ratio because they were truncated by the end of recording during E1 so it was unknown whether or not they would have produced E2) in contrast to 13 out of 40 (33%) phloem phases on Iroquois (*P* < 0.0001, Fisher's Exact Test). Initial E1 bout duration was significantly longer on TGR‐1551 than on Iroquois (22.9 ± 15.0 vs. 8.3 ± 13.2, *P* < 0.0001, Wilcoxon rank sum test, Table 1). The distribution of the E1 bout duration data was skewed with a long right hand tail for Iroquois; therefore, the medians may provide a better statistic showing the degree of difference between TGR‐1551 and Iroquois: 21.7 min and 1.1 min, respectively (Table 1).

**Table 1:** Duration of phloem phase (PP), waveform E2 within each phloem phase (scored as zero if the phloem phase did not attain E2), and waveform E1 on Iroquois and TGR‐1551 melons. Durations are in minutes. Entries are mean ± standard deviation (*n*). Probability level is from the nonparametric Wilcoxon rank sum test

Melon	PP duration	E2 duration	E1 duration
Iroquois	511.6 ± 342.0 (27)	496.9 ± 350.3 (27)	8.3 ± 13.2 (27)
	Median = 399.4	Median = 395.2	Median = 1.1
TGR‐1551	34.8 ± 49.7 (30)	12.5 ± 44.8 (30)	22.9 ± 15.0 (28)
	Median = 23.0	Median = 0	Median = 21.7
Probability	<0.0001	<0.0001	<0.0001

**Figure 1:**
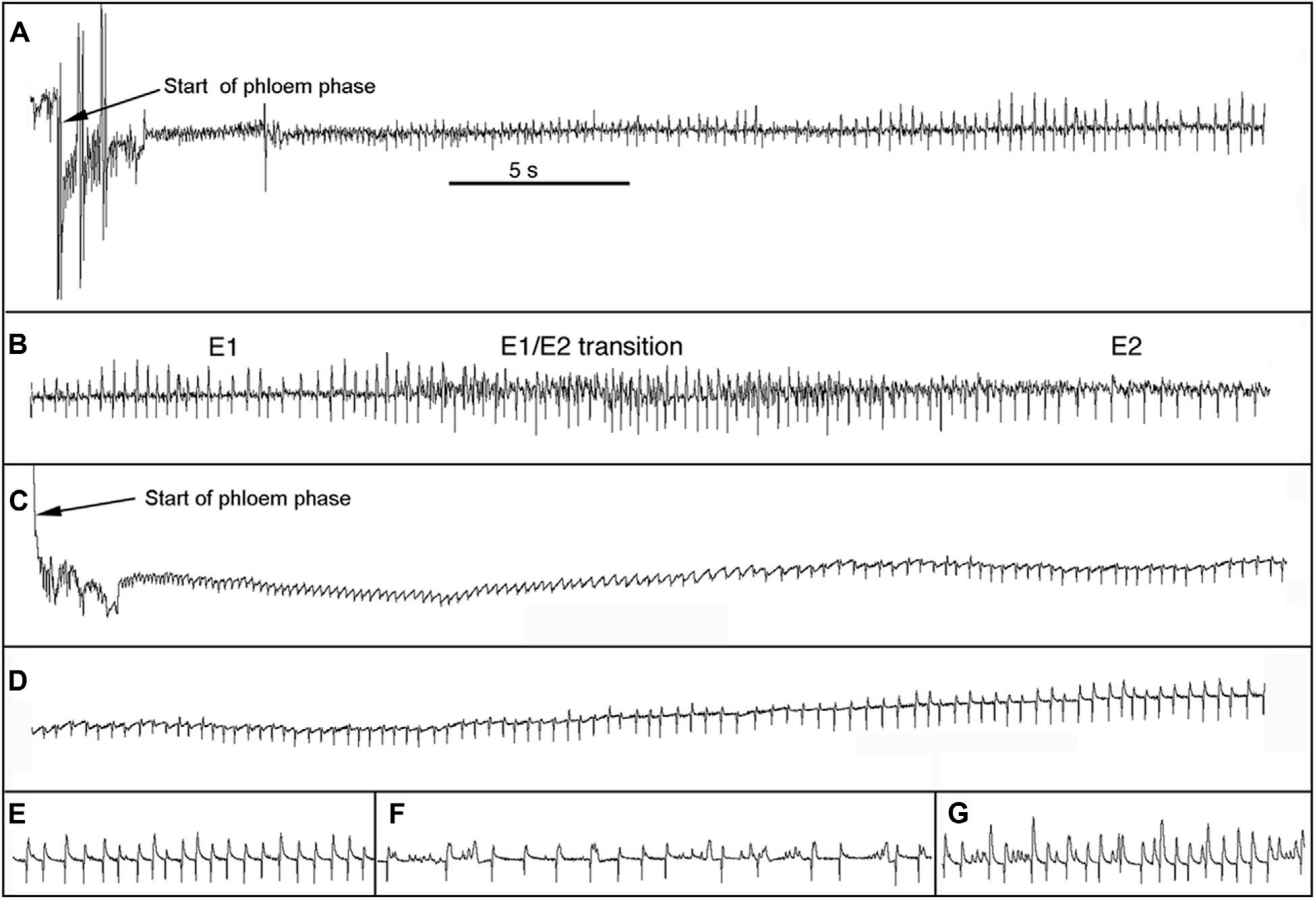
Examples of phloem phase waveforms on Iroquois (A and B; at different times in the same phloem phase; both at the same voltage and time scales) and TGR‐1551 (C–G; all at different times in the same phloem phase; all at the same voltage and time scales). The phloem phase on Iroquois (A & B) lasted > 13 h (it went beyond the end of the recording); all except the first 47 s was waveform E2. The phloem phase on TGR‐1551 (C–G) lasted 9.45 min and consisted entirely of waveform E1. All subfigures have same time scale as indicated in subfigure A. (A) Start of phloem phase on Iroquois. (B) Later in same phloem phase showing the transition from waveform E1 to E2. The E1/E2 transition began about 34 s after the start of phloem phase and E2 started about 13 s later. (C) Start of phloem phase on TGR‐1551. (D) Later in same phloem phase showing how upward spikes of E1 often develop gradually over time (there is some overlap between C and D). (E) Sample of E1 100–110 s after start of phloem phase. (F) Sample of E1 302–317 s after start of phloem phase. (G) Sample of E1 538–548 s after start of phloem phase. Phloem phase ended 19 s later.

**Figure 2:**
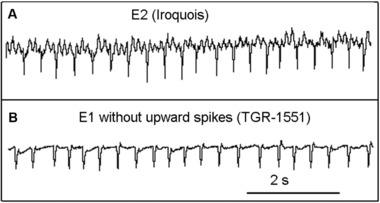
Contrasting E2 on Iroquois and E1 without upward spikes on TGR‐1551. (A) Waveform E2 on Iroquois. (B) Variation of waveform E1 without upward spikes on TGR‐1551. Both exhibit regular downward spikes, but E2 is characterized by low‐amplitude waves (2–3 between each downward spike) that are lacking in E1. Also, E2 is almost always preceded by a distinct E1/E2 transition waveform (Fig. 1B; Tjallingii, 1990; van Helden & Tjallingii, 1993) whereas E1 without upward spikes is not.

The structure of waveform E1 was not constant and evolved over time, especially during long duration E1's that were characteristic of TGR‐1551. The evolution in structure varied among phloem phases, but a general description can be made that applies to most. Immediately after the voltage drop when the stylet tips penetrated the sieve element, voltage briefly fluctuated rapidly without a discernable pattern usually for no more than 5 or 6 s (e.g., the first ≈2.5 s in Fig. 1A, C). Voltage fluctuation then usually became more regular and formed a sequence of more distinct patterns that evolved in both shape in frequency. Most commonly, the sequence began at high frequency with sharp downward spikes (e.g., next 2–3 s in Fig. 1A, C) and then sometimes gradually, sometimes abruptly, the frequency decreased. In TGR‐1551, as the frequency decreased, the pattern often began to take on a wave‐like appearance (Fig. 1C). This sequence of patterns was very variable in duration, from a few seconds to several minutes. In the great majority of cases, this sequence was followed by a pattern that occupied most of the time (usually >70%) of long E1s; and frequently lasted over 20 min on TGR‐1551. This pattern (starting about 6 s after beginning of phloem phase in Fig. 1A and after about 20 s in Fig. 1C) consisted of sharp downward spikes, similar to those seen in E2, but lacking the higher‐frequency, low‐amplitude waves characteristic of E2 (Figs. 1C, D and 2B); in addition to the downward spikes, there also were upward spikes that were sharp and distinct from the onset of this pattern (Fig. 1A) or started out small or absent and gradually became larger and more distinct (Fig. 1C, D). When the upward spikes became distinct, the waveform was more or less the same as the “standard E1” as illustrated by Tjallingii (1995, 2006). When the “standard E1” pattern was long, its amplitude often increased over time and its shape frequently changed (Fig. 1D–G). It should be stressed that this description of E1 is general and there is considerable variation. Often one pattern gradually morphed into another, making a precise description of all the variations infeasible.

### Sieve element occlusion in TGR‐1551 and Iroquois

**Figure 3:**
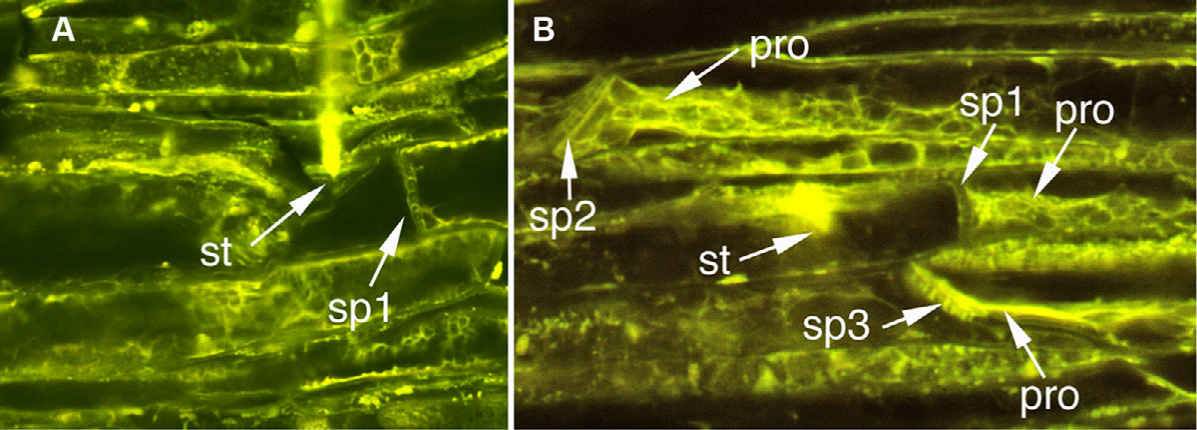
Stylet tips in sieve elements of Iroquois (A) and TGR‐1551 (B) melon. (A) Iroquois: aphid and leaf were cryofixed 165 s after initial penetration of the sieve element. Phloem phase began with 94 s of waveform E1 and sample was cryofixed during E2, 70 s after the transition from E1 to E2. Damage to the left of the stylet tips occurred during dissection. (B) TGR‐1551: aphid and leaf were cryofixed 229 s after initial penetration of the sieve element. The aphid was engaged in waveform E1 the entire 229 s. pro, protein occlusion; sp1, sieve plate of penetrated sieve element; sp2–3, sieve plates of sieve elements not penetrated by the stylet tips; st, stylet tips.

Cryofixation shortly after the start of phloem phase showed that sieve elements were almost always occluded on TGR‐1551 but not on Iroquois (Fig. 3). Twenty‐six samples from TGR‐1551 and 20 samples from Iroquois were successfully processed. The 26 samples from TGR‐1551 were cryofixed 126–1013 s after the start of phloem phase (median = 211 s) and 25 were in waveform E1 at the time of cryofixation (the recording in one sample was too noisy to distinguish E1 and E2). The 20 samples from Iroquois were cryofixed 117–677 s after the start of phloem phase (median = 196 s). Fifteen of the 20 Iroquois samples were in waveform E2 at the time of cryofixation; three samples were still in the initial E1; one sample reached E2 but returned to E1 13 s before cryofixation, and the EPG recording for one sample was too noisy to distinguish E1 and E2. In most, but not all samples, the penetrated sieve element could be identified with confocal microscopy. Sieve elements are very elongate cells and it was not always possible to trace the cell from the penetration point to the sieve plates at each end. Consequently, the samples were scored in Table 2 according to what could be observed (e.g., both sieve plates or only one sieve plate of the penetrated cell; sieve plates of sieve elements close to the stylets, etc.). Scores in rows 1–5 of Table 2 can be considered a clear occlusion response and scores in rows 9–11 can be considered a clear lack of occlusion. Twenty‐three samples from TGR‐1551 fall in the clear occlusion category and only one in the clear lack of occlusion category. In contrast, 5 samples from Iroquois fall in the clear occlusion category while 13 fall in the clear lack of occlusion category (96% occlusion for TGR‐1551 vs. 28% occlusion for Iroquois; *P* < 0.0001, Fisher's Exact Test). The five Iroquois samples in the occlusion category included two that were still in the initial E1 waveform and three that were in waveform E2 at the time of cryofixation. The 13 Iroquois samples in the nonocclusion category included 1 still in the initial E1 waveform and 12 that were in waveform E2 at the time of cryofixation.

**Table 2:** Evaluation of state of plugging of sieve plates (SPs) of the penetrated sieve elements (SEs) in TGR‐1551 and Iroquois melon. Entries are the number of samples in each score category for TGR‐1551 (total 26 samples) and Iroquois (total 20 samples) melons

Score	TGR‐1551	Iroquois
1. Both SPs of penetrated SE are plugged	9	0
2. Both SPs of the penetrated SE were identified; one is plugged; other unplugged or only partially plugged	4	3
3. One SP of the penetrated SE was identified and is plugged; other SPs that could potentially be at the other end of the SP also are plugged	4	0
4. One SP of the penetrated SE was identified and is plugged; other SP was not found	4	1
5. Cannot identify the penetrated SE; at least one SP in the area around the stylets is plugged	2	1
6. One SP of the penetrated SE was identified and appears to be partially clear with some presumed protein encroaching from a parietal position and covering about half the SP; other SP was not found	0	1
7. Stylet tips are in a companion cell (CC)—the SE associated with the CC has one plugged and one clear SP; another nearby SP of a nonpenetrated SE was plugged	1	0
8. Penetrated SE has reticulated substance on SP; another nearby SP of a nonpenetrated SE is lightly plugged	1	0
9. Both SPs of penetrated SE are clear	0	6
10. One SP of the penetrated SE was identified and is clear; the other SP was not found; no plugs were seen in the area where the other end of the SE would be located	0	5
11. Cannot positively ID the SPs of the penetrated SE but no plugs seen in the area	1	2
12. At least one SP of penetrated SE is clear; the other SP was not found; some plugged SPs in nonpenetrated cells were found	0	1

**Figure 4:**
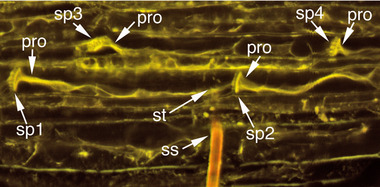
TGR‐1551 sieve elements occluded with protein. Note that even sieve elements that have not been touched by the stylets also are plugged. Sieve plates 1 and 2 (sp1 and sp2) are at either end of the penetrated sieve element; sp3 and sp4 are sieve plates from a sieve element that was not in contact with any portion of the stylets and was not in the same sieve tube as the penetrated sieve element nor were there any salivary sheaths that indicated it had been touched by the stylets prior to cryofixation. This sample was cryofixed 203 s after initial penetration of the sieve element. The aphid was engaged in waveform E1 the entire 203 s. pro, protein occlusion; ss, stylet shaft; st, stylet tips.

Interestingly, for samples in the occlusion category both for TGR‐1551 and Iroquois, the penetrated sieve element was not the only occluded sieve element seen; in most of these samples, multiple plugged sieve plates were observed in nonpenetrated sieve elements; thus the general area around the penetrated sieve element seems to be affected (Figs. 3B, 4). In some cases, occlusion was observed in sieve elements that were not in the same sieve tube as the penetrated sieve element and were not in the stylet path and thus were never contacted by the stylets (Fig. 4).

### Sieve element occlusion in lanthanum‐treated and control TGR‐1551

**Figure 5:**
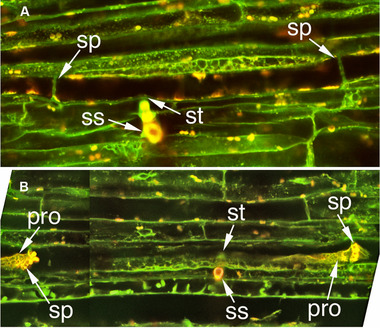
TGR‐1551 sieve elements cryofixed shortly after initial penetration of sieve element with the veins treated with either lanthanum saline or standard saline. (A) Vein treated with lanthanum saline; cryofixed 232 s after initial penetration of sieve element. Phloem phase began with 82 s of waveform E1 and sample was cryofixed during E2, 150 s after the transition from E1 to E2. (B) Vein treated with standard saline; cryofixed during waveform E1, 277 s after initial penetration of the sieve element. The aphid was engaged in waveform E1 the entire 277 s. This image is a montage of two images at the same *XY* coordinates but different focal planes to show the stylet tips and right sieve plate of the penetrated sieve element in one image and the left sieve plate in the other. pro, protein occlusion; sp, sieve plate of penetrated sieve element; ss, stylet shaft; st, stylet tips.

Cryofixation shortly after the start of phloem phase on lanthanum‐treated TGR‐1551 demonstrated that application of the calcium channel blocker, lanthanum chloride, to the midribs of TGR‐1551 had its intended effect: SEO protein occlusion did not occur in response to stylet penetration of sieve elements (Fig. 5A). Fourteen samples from lanthanum‐treated midribs and eight samples from standard saline‐treated midribs were successfully processed. The 14 samples from lanthanum‐treated midribs were cryofixed 229–334 s (median = 252 s) after the start of phloem phase. All 14 were cryofixed during waveform E2 (phloem sap ingestion) which began 30–210 s after the start of phloem phase. The eight samples from standard saline‐treated midribs were cryofixed 190–341 s (median = 241.5 s) after the start of phloem phase and all 8 were still in waveform E1 (salivation into the sieve element). Scores in rows 1–3 of Table 3 can be considered a clear occlusion response and scores in rows 4–6 can be considered a clear lack of occlusion. All 14 samples in the lanthanum treatment scored in the nonocclusion category while only 1 out of 8 samples in the standard saline treatment scored in the nonocclusion category, whereas 7 out of 8 scored in the occlusion category (Table 3).

**Table 3:** Evaluation of state of plugging of sieve plates (SPs) of the penetrated sieve elements (SEs) in the lanthanum saline versus standard saline experiment. Entries are the number of samples in each score category for lanthanum saline (total 14 samples) and standard saline (total 8 samples) treatments

Score	Lanthanum saline	Standard saline
1. Both SPs of penetrated SE are plugged and also is at least one SP of a nonpenetrated SE	0	5
2. Can identify only 1 SP of the penetrated SE and it is plugged; the other end of the penetrated SE was obliterated in dissection	0	1
3. Stylet tips were in a large SEO protein mass, the mass only partially covers the SP; the other SP of the penetrated SE is somewhat similar: there is a large SEO protein mass that barely touches it	0	1
4. Both SPs of penetrated SE are clear; no plugs detected in the area around the stylets	10	1
5. At least one SP of penetrated SE is clear (the other SP was not found); no plugs detected in the area around the stylets	3	0
6. Cannot positively ID the SPs of the penetrated SE but all potential SPs are clear and no plugs seen in the area around the stylets	1	0

### Phloem phase behavior on lanthanum‐treated and control TGR‐1551

When midribs of TGR‐1551 were treated with lanthanum to prevent SEO protein occlusion, aphids readily engaged in long bouts of phloem phase and E2 (Table 4). Twenty‐five phloem phases from 17 aphids feeding on lanthanum‐treated midribs and 53 phloem phases from 23 aphids feeding on control midribs were recorded. Phloem phase bout duration was significantly greater on lanthanum‐treated midribs compared to the controls (348.6 ± 201.8 vs. 45.6 ± 59.6, *P* < 0.0001, Wilcoxon rank sum test, Table 4). Similarly, the duration of sieve element ingestion (waveform E2) per phloem phase also was much greater for aphids in the lanthanum treatment than for those in the control treatment (344.7 ± 205.4 vs. 19.4 ± 49.6, *P* < 0.0001, Wilcoxon rank sum test, Table 4). The distribution of the E2 data for the control treatment had a long right hand tail; therefore, the medians may provide a better statistic showing the degree of difference between the control and lanthanum treatment: 0 min and 394.6 min, respectively (Table 4). For 17 out of 23 aphids (74%) in the control, none of the phloem phases produced waveform E2 in contrast to only 2 out of 17 aphids (12%) in the lanthanum treatment (*P* = 0.0001, Fisher's exact Test). Considering all phloem phase bouts (rather than compiled per insect), 44 out of 53 phloem phases (83%) on the control treatment did not produce E2 in contrast to 10 out of 25 (40%) on the lanthanum treatment (*P* = 0.0002, Fisher's Exact Test). Initial E1 bout duration on TGR‐1551was significantly shorter on lanthanum‐treated veins compared to standard saline‐treated (control) veins (3.9 ± 6.5 vs. 25.5 ± 30.1, *P* = 0.0009, Wilcoxon rank sum test, Table 4).

**Table 4:** Duration of phloem phase (PP), waveform E2 within each phloem phase (scored as zero if the phloem phase did not attain E2), and waveform E1 on lanthanum‐treated and control TGR‐1551 melons. Durations are in minutes. Entries are mean ± standard deviation (*n*). Probability level is from the nonparametric Wilcoxon rank sum test

Treatment	PP duration	E2 duration	E1 duration
Lanthanum	348.6 ± 201.8 (17)	344.7 ± 205.4 (17)	3.9 ± 6.5 (17)
	Median = 395.8	Median = 394.6	Median = 1.2
Control	45.6 ± 59.6 (23)	19.4 ± 49.6 (23)	25.5 ± 30.1 (23)
	Median = 17.4	Median = 0	Median = 17.4
Probability	<0.0001	<0.0001	0.0009

## Discussion

### Sieve element occlusion inhibits phloem sap ingestion in TGR‐1551

Feeding behavior on TGR‐1551 compared to a susceptible melon using this study's North American strain of *A. gossypii* was very similar to that observed by Garzo *et al*. (2002) and Tjallingii (2006) using a European strain of *A. gossypii*: ingestion (E2) was greatly reduced, bouts of phloem salivation (E1) were much longer on TGR‐1551, and the proportion of phloem phases that did not result in ingestion was much greater on TGR‐1551. Garzo *et al*. (2002) hypothesized that phloem occlusion in TGR‐1551 was responsible for the difficulty experienced by aphids attempting to ingest phloem sap from TGR‐1551, and our EPG results led us to the same hypothesis.

Cryofixation of the phloem shortly after stylet penetration of a sieve element and subsequent microscopic examination of phloem supported this hypothesis. Ninety‐six percent of penetrated TGR‐1551 sieve elements were occluded in contrast to only 28% of penetrated Iroquois sieve elements. Additional support for this hypothesis was provided by Garzo *et al*. (2018). In that study, *A. gossypii* stylets were severed by stylectomy during phloem phase and the tissue was subjected to slow, gentle chemical fixation followed by examination with transmission electron microscopy. They found coagulated protein in the food canal of *A. gossypii* feeding on TGR‐1551 but not aphids feeding on the susceptible melon cultivar, Regal. They also found protein deposits around the maxillary stylet tips in the penetrated sieve elements of TGR‐1551 but not in the susceptible melon.

The presence of protein coagulation occluding sieve elements (this study) as well as in the food canal and around the maxillary stylet tips (Garzo *et al*., 2018) when aphids fed on TGR‐1551 and the low frequency of occlusion when aphids fed on susceptible melon cultivars demonstrate that protein occlusion is associated with inhibition of phloem sap ingestion on TGR‐1551. The lanthanum experiments provide evidence that the relationship is causal, not merely correlative. When phloem was treated with the calcium channel‐blocker lanthanum, sieve element penetration by the aphids did not trigger occlusion, and the aphids were able to engage in long bouts of phloem sap ingestion.

### Sieve element occlusion may be a widespread mechanism of plant resistance against phloem sap feeders

This study is the second to demonstrate that when occlusion by sieve element protein is prevented, aphids readily feed on a plant on which they normally have difficulty feeding. When the generalist aphid, *Myzus persicae* Sulzer feeds on *Vicia faba*, forisomes (SEO protein bodies in Fabaceae) react by occluding the sieve elements and the aphids have the same difficulty ingesting sap as observed in the present study for *A. gossypii* feeding on TGR‐1551: few phloem phases achieve ingestion, and E1 periods are greatly extended (Medina‐Ortega & Walker, 2015). However, when *V. faba* phloem is treated with lanthanum, sieve element occlusion is prevented and *M. persicae* readily ingests phloem sap. Thus, SEO protein occlusion may be a widespread mechanism of resistance, and the many other aphid/plant combinations in the literature where aphids exhibit difficulty ingesting sap after penetrating a sieve element would be promising systems for similar studies.

The ability of phloem protein occlusion to interfere with sap ingestion by aphids has also been demonstrated by experimentally triggering phloem occlusion while aphids are ingesting phloem sap and then observing the aphid response. Phloem occlusion can be triggered by a burn stimulus at the distal end of a vein in melon, pumpkin, and faba bean (and presumably other plant species). The burn stimulus initiates an electropotential wave that propagates basipetally through the phloem, triggering sieve element occlusion as the wave passes (Furch *et al*., 2007, 2010; Walker, unpublished data). At least seven aphid species feeding on the veins of four plant species respond to a burn stimulus at the distal end of a vein by switching from phloem sap ingestion (waveform E2) to salivation (waveform E1) (Will *et al*., 2007, 2009; Furch *et al*., 2010; Medina‐Ortega & Walker, 2013) (additionally, an eighth aphid species [*A. gossypii*] on a fifth plant species [*C. melo*] and a whitefly [*Bemisia argentifolii* Bellows & Perring] on *Vicia faba* also respond in the same way to experimentally induced phloem occlusion—Walker, unpublished observations). Consequently, the ability of phloem occlusion to interfere with phloem sap ingestion is well established. This study and Medina‐Ortega and Walker (2015) show that a plant's natural phloem occlusion response to aphid feeding (rather than experimentally induced) can greatly impede phloem sap ingestion and serve as a mechanism of aphid resistance.

### Cucurbit phloem proteins and sieve element occlusion

The first proteins suspected to be involved in sieve element occlusion were two phloem proteins, PP1 and PP2 in *Cucurbita* (Cucurbitaceae) (Clark *et al*., 1997). PP1 is a filamentous protein that in response to oxidation transforms from a soluble form to a gel (Kleinig *et al*., 1971, 1975; Walker & Thaine, 1971; Walker, 1972; Read & Northcote, 1983; Alosi *et al*., 1988). PP2 is a lectin that binds to the PP1 filaments (Read & Northcote, 1983; Smith *et al*., 1987). PP1 seems to occur exclusively in *Cucurbita*, and homologs of PP1 were not detected in *Cucumis* (Clark *et al*., 1997). More recent studies have identified a large and widespread gene family of sieve element occlusion (SEO) proteins that are likely ubiquitous in dicotyledon plants, including *Cucurbita* and *Cucumis* (Ernst *et al*., 2011, 2012). The SEO proteins are not related to PP1 and PP2 (Rüping *et al*., 2010; Ernst *et al*., 2012). Both PP1 and SEO protein have been detected in *Cucurbita* sieve element plugs (Clark *et al*., 1997; Ernst *et al*., 2012); however, with no known homolog of PP1 in *Cucumis*, the plugs observed in this study are probably formed by SEO proteins. The triggering mechanism for formation of SEO protein plugs so far has been studied only for forisomes, the distinctive SEO protein bodies in legumes. Occlusion by forisomes is triggered by an influx of calcium into the sieve element (Knoblauch *et al*., 2001; Furch *et al*., 2009); however, a mechanism for triggering occlusion by nonforisome SEO proteins has not yet been published. The ability of the calcium channel blocker, lanthanum, to prevent sieve element protein plugs in TGR‐1551 suggests that a calcium influx also triggers plugging by a nonforisome SEO protein, although other potential effects of lanthanum cannot be ruled out.

### Possible role of callose inhibiting sap ingestion?

It has been proposed that phloem occlusion is a two‐stage process: rapid occlusion by proteins followed by a slower developing constriction of sieve pores by callose deposition (van Bel, 2006). The present study demonstrates that sieve element occlusion by proteins in melon is prevented by trivalent lanthanum, a calcium channel blocker. Callose deposition in sieve pores is triggered by an influx of calcium into the sieve element (Fredrikson & Larsson, 1992) and therefore would also be expected to be inhibited by lanthanum. We did not test for callose deposition in our microscopy study; consequently, a role for callose in prevention of sap ingestion cannot be ruled out. Sieve element occlusion by SEO proteins is a much more rapid response than callose deposition in the sieve pores (Furch *et al*., 2007, 2010) and so SEO proteins seem more likely than callose to be effective at occlusion at the time when aphids would otherwise make the transition from E1 salivation to E2 ingestion which is a little over 1 min after the start of phloem phase (on Iroquois, median E1 duration = 1.1 min, Table 1). Callose deposition in *Cucurbita maxima*, *Solanum lycopersicum* and *Vicia faba* is just starting to accumulate 1.1 min after a burn stimulus (Furch *et al*., 2007, 2010). Therefore, if calcium influx into a TGR‐1551 sieve element is triggered at the beginning of phloem phase, as opposed to earlier in the feeding process, then it seems likely that protein occlusion is mainly responsible for inhibition of phloem sap ingestion in this study. However, it is unknown when the calcium influx is initiated when *A. gossypii* feeds on TGR‐1551. The role of deposition of callose in sieve pores in inhibition of ingestion could be investigated independent of sieve element proteins by employing the callose synthesis inhibitor 2‐deoxy‐D‐glucose (Fredrikson & Larsson, 1992) or by gene silencing (Zhai *et al*., 2017). A further caveat when interpreting the results of the lanthanum experiments: calcium is a ubiquitous physiological signal involved in many plant functions and lanthanum is known to have other effects on plant physiology including the jasmonic acid defense pathway (Zhou *et al*., 2012). Nonetheless, the demonstration that experimentally induced protein occlusion of sieve elements quickly stops phloem sap ingestion by aphids (Will *et al*., 2007, 2009; Furch *et al*., 2010; Medina‐Ortega & Walker, 2013) suggests that the protein occlusion observed in this study is, by itself, capable of preventing phloem sap ingestion from TGR‐1551.

### What triggers occlusion in TGR‐1551?

In both TGR‐1551 fed on by *A. gossypii* and faba bean fed on by *M. persicae* (Medina‐Ortega & Walker, 2015), phloem occlusion was not limited to the penetrated sieve element (Figs. 3B, 4 and fig. 2 in Medina‐Ortega & Walker, 2015). Occlusion occurred both upstream and downstream of the penetrated sieve element and in sieve elements that were not part of the same sieve tube as the penetrated sieve element (i.e., parallel to but laterally separated from the penetrated sieve element). Additionally, occlusion also was frequently observed in sieve elements that were not touched by the stylet path and thus could not have been penetrated during the pathway phase preceding phloem phase (sieve element penetration sometimes occurs during pathway phase and produces only a potential drop (pd) waveform rather than the characteristic phloem phase waveforms [Tjallingii & Hogen Esch, 1993]). So what is the trigger for occlusion? The distribution of occlusion argues against a direct effect of saliva injected into the penetrated sieve element. Saliva secreted in the sieve element would be expected to affect the penetrated sieve element and possibly downstream sieve elements in the same sieve tube, but not upstream sieve elements and sieve elements from different sieve tubes. Medina‐Ortega and Walker (2015) proposed two hypotheses to explain the occlusion in nonpenetrated sieve elements that were not in the stylet path: (1) As the stylets approach the phloem, saliva secreted extracellularly (Moreno *et al*., 2011) diffuses through the apoplast triggering sieve element occlusion in the general vicinity, probably by activating calcium channels and (2) the penetrated sieve element, in response to either mechanical penetration or injected saliva, generates an electropotential wave that propagates in all directions from the penetrated sieve element, activating voltage‐gated calcium channels and triggering occlusion (van Bel *et al*., 2014). The second hypothesis could also be extended to non‐sieve element cells in the phloem that may be punctured and recorded as potential drops (pds) in the EPG recordings.

### Potential role of aphid saliva in overcoming sieve element occlusion

In an experiment by Will *et al*. (2007), aphid saliva concentrated from artificial diet fed upon by over 6000 aphids reversed the occlusion response of faba bean forisomes (SEO protein bodies in Fabaceae) *in vitro*. However, in an *in vivo* experiment with the natural situation of a single aphid feeding on a sieve element, salivation was unable to reverse occlusion by forisomes (Medina‐Ortega & Walker, 2013). Assuming that all the variations of waveform E1 in Fig. 1 represent salivation into the sieve element, this study on TGR‐1551 also suggests that salivation is ineffective at reversing sieve element occlusion. In the cryofixation study, three samples of TGR‐1551 were cryofixed more than 10 min after the start of phloem phase during which only waveform E1 was recorded and all three were occluded including one sample where the aphid engaged in E1 for 16.9 min before cryofixation. The long durations of E1 on TGR‐1551 (Table 1), the great majority of which were not followed by E2, also suggest that salivation is not effective at reversing SEO protein occlusion. If saliva plays a role in overcoming sieve element occlusion in compatible aphid‐plant combinations (where aphids ingest phloem sap without any apparent difficulty), its role would likely be preventing the occlusion response from occurring in the first place rather than reversing occlusion after it occurs.

### Aphid behavior preceding sap ingestion in phloem phase is complex and not well understood

Finally, phloem phase traditionally has been considered to consist of two waveforms, E1, which has been correlated with secretion of watery saliva into the sieve element, and E2, which has been correlated with ingestion of phloem sap and concurrent salivation (Tjallingii, 1995). In this traditional view, phloem phase always starts with E1, and depending on whether or not phloem phase is successful (i.e., achieves sap ingestion), E1 may or may not be followed by E2. Examples of E1 appear in Tjallingii (1990, 1995, 2006) and Garzo *et al*. (2018), and many other publications from Tjallingii and collaborators. These published examples are generally used as the standard for comparison when identifying E1 in EPG recordings, and in general are similar to the waveforms in the right half of Fig. 1A, left half of Fig. 1B, right side of Fig. 1D, and Fig. 1E–G. Recently, Garzo *et al*. (2018) noted that in contrast to the traditional view, phloem phase does not start with standard E1, but rather E1 begins 10–20 s after a sieve element is punctured, and the waveform during the initial 10–20 s period is similar to the waveform during a pd. In this study, multiple waveform shapes precede “standard E1” on both Iroquois and TGR‐1551 (Fig. 1) and this can considerably delay the onset of “standard E1,” especially on TGR‐1551. One variation of E1 seen primarily on TGR‐1551 superficially resembles waveform E2 (e.g., right side of Fig. 1C, left side of Fig. 1D and Fig. 2B) due to the prominent downward spikes and absence of upward spikes; however, this variation lacks the lower‐amplitude, higher‐frequency waves that occur between the downward spikes in waveform E2 (Fig. 2A). It is these low‐amplitude, high‐frequency waves during E2 that are associated with ingestion (Tjallingii, 1987, 1990). The dominant downward spikes are not associated with ingestion as they do not correspond to cibarial pump activity; rather, they are associated with salivary pump activity (Tjallingii, 1978). Additionally, E2 is almost always preceded by a distinct E1/E2 transition waveform (Fig. 1B; Tjallingii, 1990; van Helden & Tjallingii, 1993) whereas E1 without upward spikes is not preceded by such a transition and it usually gradually morphs into a “standard E1” waveform as upward spikes become more pronounced (e.g., Fig. 1D).

The underlying concept that makes EPG so useful for studying feeding behavior of aphids and other piercing‐sucking insects is that differences in behavior generate differences in waveforms, potentially allowing researchers to detect as many different behaviors as there are different waveforms (Walker, 2000). Consequently, the variety of waveforms seen during phloem phase in this study indicates that a lot more behavioral variation is occurring during E1 than simply secretion of watery saliva into the sieve element. E1 behavior is likely to play a major role in whether or not successful ingestion will occur. Consequently, elucidation of the behavioral correlations of these waveform variations may prove very valuable for enhancing the understanding of aphid–plant interactions.

## Disclosure

The authors declare that the research was conducted in the absence of any commercial or financial relationships that could be construed as a potential conflict of interest.
